# High proportion of post-migration HIV acquisition in migrant men who have sex with men receiving HIV care in the Paris region, and associations with social disadvantage and sexual behaviours: results of the ANRS-MIE GANYMEDE study, France, 2021 to 2022

**DOI:** 10.2807/1560-7917.ES.2024.29.11.2300445

**Published:** 2024-03-14

**Authors:** Romain Palich, Andrés Arias-Rodríguez, Martin Duracinsky, Jean-Yves Le Talec, Olivia Rousset Torrente, Caroline Lascoux-Combe, Karine Lacombe, Jade Ghosn, Jean-Paul Viard, Gilles Pialoux, Michel Ohayon, Claudine Duvivier, Annie Velter, Mohamed Ben Mechlia, Lydie Beniguel, Sophie Grabar, Maria Melchior, Lambert Assoumou, Virginie Supervie, GANYMEDE study group, Collaborators, Fabienne CABY, Juliette GERBE, Laurence COURDAVAULT, Elisabete GOMES, Carole LOUISIN, Fatima TOUAM, Elise GARDIENNET, Françoise CHURAQUI, Carolie PALLIER, Johann CAILHOL, Flory MFUTILA KAYKAY, Sonia OULD YOUNES, Christia PALACIOS, Hugues CORDEL, Héloïse DELAGREVERIE, Youssouf MOHAMED KASSIM, Nicolas VIGNIER, Anaenza FREIRE-MARESCA, Marina KARMOCHKINE, Alice Andrée MARIAGGI, Dominique SALMON, Valérie LE BAUT, Marie-Pierre PIETRI, Myriam KALAMBAY, Marie-Josée DULUCQ, William VENDRIOS, Raida BENRAYANA, Magali BOUVIER ALIAS, Romain PALICH, Christine KATLAMA, Yasmine DUDOIT, Naoual QATIB, Cathia SOULIE, Elisa TEYSSOU, Raynald FELIHO, Thibault CHIARABINI, Oumarou NABI, Bénédicte LEFEVRE, Nadia VALIN, Julie LAMARQUE, Ouazene ZINEB, Diane BOLLENS, Djeneba FOFANA, Skander BENOTHMANE, Miresta PREVILON, Fernando IGLESIAS SANCHEZ, Marie-Laure CHAIX BAUDIER, Antoine BACHELARD, Olivia DA CONCEICAO, Françoise LOUNI, Sylvie LE GAC, Manuella ONAMBELE, Mélanie BERTINE, Fella MAZOUZ, Elisabeth ROUVEIX, Soumia CHENAKEB, Frédérique MOREAU, Abdessamad KACHAL, Fadia HAMROUNI, Inès MARAGHNI, Nassima BOUMGHAR, Dehbia BENKERROU, Serge RODRIGUES, Aurore DURAND, Julien HUYARD, Frédérique THONON, Pascal BESSONNEAU, Guillaume ROUCOUX, Véronique DORE, Cyriac BOUCHET, Kostia LENNES

**Affiliations:** 1 Sorbonne University, Pitié-Salpêtrière hospital, AP-HP, Paris, France; 2 Sorbonne Université, Pierre Louis Epidemiology and Public Health institute (iPLESP), INSERM 1136, Paris, France; 3 Paris Cité University, Patient-Reported Outcomes Unit (PROQOL), INSERM 1123, Paris, France; 4 Toulouse Jean Jaurès University, CERTOP, CNRS UMR 5044, Toulouse, France; 5 Paris Cité University, Saint Louis hospital, AP-HP, Paris, France; 6 Sorbonne University, Saint Antoine hospital, AP-HP, Paris, France; 7 Paris Cité University, Bichat hospital, AP-HP, Paris, France; 8 Paris Cité University, Hôtel-Dieu hospital, AP-HP, Paris, France; 9 Sorbonne University, Tenon hospital, AP-HP, Paris, France; 10 Le 190 Sexual Health Centre, Paris, France; 11 Paris Cité University, Necker hospital, AP-HP; INSERM U1016, CNRS UMR8104, Institut Cochin; IHU Imagine; Institut Pasteur Medical Center, Paris, France; 12 Public Health France (SPF), Paris, France; 13 French National Agency for Research on AIDS, Viral Hepatitis and Emerging Infectious Diseases (ANRS-MIE), Paris, France; 14 The members of the GANYMEDE study group are listed under Collaborators

**Keywords:** HIV, migrants, men who have sex with men, post-migration, foreign-born, born abroad

## Abstract

**Background:**

Some migrant men who have sex with men (MSM) acquire HIV in France.

**Aims:**

We investigated, in migrant MSM receiving HIV care in France, the (i) rate of post-migration-HIV acquisition in France, (ii) delay between arrival and HIV acquisition and (iii) factors affecting HIV acquisition within 1 year after migration.

**Methods:**

This cross-sectional study focused on ≥ 18-year-old MSM born outside France, receiving HIV care in the Paris region. Information on migration history, socioeconomic condition, sexual activity, and health was collected in May 2021–June 2022 through self-administered questionnaires and medical records. Post-migration-HIV-acquisition rate and delay between arrival in France and HIV acquisition were estimated from biographical data and CD4^+^ T-cell counts. Predictors of HIV acquisition within 1 year after migration were determined using logistic regression.

**Results:**

Overall post-migration HIV-acquisition rate was 61.7% (715/1,159; 95%CI: 61.2–62.2), ranging from 40.5% (95%CI: 39.6–41.6) to 85.4% (95%CI: 83.9–86.0) in participants from Latin America and North Africa. Among post-migration-HIV acquisitions, those within 1 year after migration represented 13.1% overall (95%CI: 11.6–14.6), being highest in participants from sub-Saharan Africa (25%; 95%CI: 21.5–28.3). Participants ≥ 15-years old at migration, with post-migration-acquired HIV, had a 7.5-year median interval from arrival in France to HIV acquisition (interquartile range (IQR): 3.50–14.75). Older age at arrival, region of origin (sub-Saharan Africa and Asia), degree of social disadvantage and numbers of sexual partners were independently associated with acquiring HIV within 1 year in France.

**Conclusion:**

Our findings may guide HIV prevention policies for most vulnerable migrants to Europe.

**Key public health message**

**What did you want to address in this study and why?**

The GANYMEDE study meant to estimate, among foreign-born men who have sex with men (migrant MSM), the proportion who acquired HIV after migrating to France. Among migrant MSM who acquired HIV after migration, the study also aimed to identify factors associated with acquiring HIV within the first year after arrival in the country. Participants in this study were ≥ 18-year-old MSM with HIV, treated in the Paris region, and born outside of France.

**What have we learnt from this study?**

Post-arrival, ca 62% of infected migrant MSM had acquired HIV in France, 13% of whom within the first year. Among study participants, 35% reported leaving their country of birth due to sexual-orientation related issues. Younger age at arrival, certain regions of origin, e.g. sub-Saharan Africa or Asia, higher social disadvantage level and higher numbers of sexual partners were independently associated with acquiring HIV during the first year in France.

**What are the implications of your findings for public health?**

Contextual e.g. legal status of homosexuality in the country of birth, and individual factors e.g. level of social disadvantage and sexual behaviour, may contribute to early HIV acquisition post migration. Our findings highlight the need for HIV prevention services to reach the most vulnerable migrants in western European countries.

## Introduction

Currently in most western European countries, almost half of all people living with HIV (PLWH) are migrants, who were not born in the country in which they reside [1]. Many countries consider migrants as a priority population in their national response to HIV. It has long been assumed that most HIV infections among migrants in Europe, particularly those from sub-Saharan Africa (SSA), are imported. Since the 2010s however, research has shown that a substantial proportion of these HIV infections are acquired after migration [2-6]. In 2017, a collaborative study, including nine European Union/European Economic Area (EU/EEA) countries, which France did not participate in, showed that the rate of post-migration HIV acquisition differed across population subgroups (72% among men who have sex with men (MSM) vs 51–58% among heterosexual individuals), host countries and geographic areas of origin (52% among individuals from sub-Saharan Africa vs 71% among those from Europe or Latin America) [7]. Knowing whether HIV acquisition occurs before or after migration is critical for designing appropriate HIV prevention and testing strategies.

HIV acquisition, whether in the country of origin or in the host country, should be considered in the context of life course, in which the migration process is a major event. Contextual factors, such as economic level, healthcare system efficiency, HIV prevalence, or legal status and social tolerance of homosexuality in the country of origin, and individual factors such as having multiple sexual partners, condomless sex, access to prevention, drug use, sex work, engagement with the gay scene, density of sexual network, may explain the increased risk of HIV exposure among migrant MSM [8,9]. Several studies carried out between 2010 and 2020 have shown that hardship during migration and in the first years after arrival in the host country influence emotional insecurity and risk taking with regards to HIV acquisition [10-13].

Since the mid-2010s, migrant MSM are known to be the population subgroup most affected by the HIV epidemic in France, as in many European countries, in terms of diagnosed and undiagnosed prevalence and incidence [14]. While new infections have been declining for several years in all population subgroups, they continue to increase among MSM born outside of France [15]. In 2018, it was estimated that while the Paris region (Île-de-France) hosts 19% of the French population, almost 50% of MSM migrants with undiagnosed HIV in France live in this region [14].

To gain more insight on HIV acquisition in migrant MSM, the ANRS-MIE 14058 GANYMEDE study recruited migrant MSM followed up for HIV in the Paris region, in France. The objectives of the study were: (i) to estimate the proportion of post-migration HIV acquisition in this population, (ii) to estimate the time between arrival in France and HIV acquisition among participants who acquired the virus after migration, (iii) to describe the reasons for migration and living conditions upon arrival in France, and (iv) to investigate factors associated with HIV acquisition within the first year in France.

## Methods

### Study design, setting and participants

We designed a multicentre, cross-sectional study among a sample of migrant MSM living with HIV in the Paris region. Participants were cisgender men (≥ 18 years old), born outside France, self-reporting having sex with other men (either currently or historically), and followed for HIV treatment in one of the 14 centres participating in the study, as described in Supplementary material, Figure S1. We aimed to include 1,200 participants, to compare individuals from different places of origin.

Data on migration history, socioeconomic conditions, sexual activity, health before, after and at the time of migration to France, were collected through a self-administered questionnaire and medical records. The questionnaire (148 items, including 62 depending on previous responses) was built after conducting an exploratory qualitative survey, based on 13 interviews among migrant MSM living with HIV, in order to identify areas of focus and refine survey language [16].

Participants gave consent to participate and to complete a 40-min-long digital questionnaire in one of the six available languages (French, English, Spanish, Portuguese, Arabic or Russian). An interpreter was available by telephone for participants who could not speak any of the six languages. Participants with the greatest difficulty in logging on or understanding the questionnaire were assisted by the research team. Only participants who immigrated after the age of 15 years completed the questionnaire, on the assumption that those who had immigrated during childhood had not begun their sexual lives and tended to have living conditions more similar to those of people born in France. Participants who immigrated before or at the age of 15 years old were included in the study but did not complete the questionnaire. Demographic and HIV-related clinical and biological data were collected for all participants. The study was offered to all patients who met the inclusion criteria. In case of refusal, age and country of birth were collected anonymously to adjust the analyses. Data were collected between May 2021 and June 2022.

### Statistical analysis

The likely country of HIV acquisition was determined based on data from questionnaires and medical records. We assumed that all participants who arrived in France before the age of 15 years acquired HIV in France, after verifying that there were no cases of mother-to-child transmission of HIV in this subgroup. For participants who arrived in France after the age of 15 years, we concluded that they acquired HIV before migration if they reported to be aware of their positive HIV status before migration, with a year of HIV diagnosis or antiretroviral therapy (ART) initiation before the year of arrival in France. Participants who did not meet the aforementioned criteria were classified as having acquired HIV after migration if they met any of the following criteria: (i) first sexual intercourse in France (self-questionnaire), (ii) at least one negative HIV test in France (self-questionnaire), or (iii) diagnosis of primary infection at least 1 year after arrival in France (medical records).

If none of these criteria were met, we used a seroconversion model using CD4+ T-cell count data to estimate the time of HIV acquisition. We built our model using data from the French Hospital Database on HIV (ANRS CO4 FHDH), which is a large hospital-based cohort, established in 1989 [17], and providing information on HIV seroconversion and CD4+ T-cell counts before ART initiation. We used non-Markovian stochastic chains with memory of variable length model assuming a retrospective increase in CD4+ T-cell count until reaching the date of the known HIV seroconversion [18]. Using this model, by integrating in it the first CD4+ T-cell count available at entry into care in France, as well as the date of arrival in France for each participant, we were able to estimate whether HIV acquisition occurred before or after migrating, and time between migration and HIV acquisition in France for participants with post-migration HIV acquisition, as detailed in the Supplementary material.

We then investigated potential predictors of HIV acquisition within the first year after arrival in France among individuals who acquired HIV after migration. Firstly, we conducted univariate logistic regression models to select the variables of interest. We then used a multiple correspondence analysis (MCA) to explore the relationship and the associations between different categories of potential explanatory variables. This revealed a set of variables, including participants' administrative/legal status, health coverage, employment situation, and financial well-being, which were closely linked. To address any potential issues of multicollinearity, we constructed an indicator of social disadvantage, using the aforementioned variables. Each variable was transformed into an ordinal variable with three levels, where level 3 represented the most precarious conditions such as irregular administrative/legal status, lack of medical coverage, unemployment or irregular employment, and insufficient economic resources; the social disadvantage indicator was derived by summing the contributions from each variable, resulting in a range of scores from 4 to 12. Individuals with an indicator score equal to or greater than 9 were classified as being disadvantaged as detailed in the Supplementary material. In the multivariate logistic regression, the social disadvantage indicator was included as an explanatory variable along with other variables including age at arrival in France, place of birth, and variables related to sexual behaviour.

## Results

### Study population

A total of 1,282 patients were approached to participate in the study, and 1,159 consented and were included, leading to a study acceptance rate of 90.4%. Among the 994 participants who migrated to France after the age of 15 years, 831 completed the questionnaire, yielding a completion rate of 83.6%. Languages used to complete the questionnaire were: French (622; 74.8%), Spanish (83; 10.0%), English (72; 8.7%), Portuguese (41; 4.9%), Arabic (12; 1.4%) and Russian (1; 0.1%). Of participants who completed the questionnaire, 472 (56.8%) did this on site and 359 (43.2%) at home; 98 (11.8%) needed assistance from the local research team to log in and answer the questionnaire, but no interpreters were requested. Participants had been in France for a median of 15 years (interquartile range (IQR): 6–32) at the time of questionnaire completion.

The geographical areas of origin of participants could be derived from their reported country of birth. European countries were in the World Health Organization (WHO) European Region. Among the 1,159 participants in the study, the three most represented geographical areas of origin were Latin America (336; 29.0%), Europe (244; 21.1%) and North Africa (187; 16.1%) (Table 1). Participants had arrived in France at a median age of 25 years (IQR: 20–31); median age at arrival in France was lowest among participants from North Africa (20 years; IQR: 10–28), and highest among those from South America (28 years; IQR: 24–33). All were cisgender men who reported having had sex with other men during their lifetime. Their median age at the time of the survey was 43 years (IQR: 34–56). All received ART, and 1,003/1,076 (93.2%) had a last HIV plasma viral load < 50 copies/mL.

**Table 1:** Participants’ demographics and HIV-related characteristics, Île-de-France, France, May 2021–June 2022 (n = 1,159)

Characteristic	All participants (n = 1,159)	Participants who completed the questionnaire (n = 831)
	Value	Value
Age in years at study inclusion, median (IQR)	43 (34–56)	**43 (34–55)**
Place of birth, n (%)		
Latin America	336 (29.0%)	275 (33.1%)
Europe	244 (21.1%)	175 (21.1%)
North Africa	187 (16.1%)	114 (13.7%)
Sub-Saharan Africa	180 (15.5%)	119 (14.3%)
Asia, Oceania	174 (15.0%)	116 (14.0%)
North America	38 (3.3%)	32 (3.9%)
Age in years at arrival in France, median (IQR)	25 (20–31)	27 (23–32)
Age at arrival in France, n (%)		
< 15 years	165 (14.2%)	0 (0.0%)
15–20 years	143 (12.3%)	121 (14.6%)
21–25 years	282 (24.3%)	229 (27.6%)
26–30 years	259 (22.3%)	216 (26.0%)
> 30 years	310 (26.7%)	265 (31.9%)
Time in years from first HIV medical visit in France, median (IQR)	5.9 (2.8–11.8)	5.4 (2.6–11.1)
Time in years from HIV diagnosis, median (IQR)	8.9 (5.1–18.1)	8.5 (4.9–16.6)
Hepatitis B co-infection (positive AgHBS), n (%)	63 (5.4%)	46 (5.5%)
Hepatitis C co-infection (positive anti-HCV Ab), n (%)	84 (7.2%)	58 (7.0%)
AIDS-event at care entry^a^, n (%)	65 (5.6%)	45 (5.4%)
First CD4+ T-cell count (in cells/mm^3^) available in France, median (IQR)	400 (246–604)	401 (250–609)
First plasma viral load available in France, n (%)		
≥ 50 copies/mL	861 (74.3%)	609 (73.3%)
< 50 copies/mL	168 (14.5%)	125 (15.0%)
Missing	130 (11.2%)	97 (11.7%)

AIDS: acquired-immunodeficiency syndrome; IQR: interquartile range.

^a^ Opportunistic disease diagnosed in the 6 months before or after the first HIV medical visit in France.

### Proportion of post-migration HIV acquisition

Based on data from questionnaires and medical records, we were able to determine whether HIV acquisition occurred before or after arriving in France for 561 of 994 individuals (56.4%) who immigrated after the age of 15 years as shown in the Supplementary material, Figure S2. Among these participants, HIV acquisition before migration was definite for 250, including 221 who had started ART before migration and 29 who had had an HIV diagnosis in a country other than France, prior to the year of arrival in France. HIV acquisition after migration was definite for 311 participants, including 282 who previously had a negative HIV test in France, and/or 78 with a documented primary HIV infection in France, and/or 52 who had had their first sexual intercourse in France. We also considered that the 165 participants who immigrated before the age of 15 years had contracted HIV in France. 

Among the 433/994 (43.6%) participants who immigrated after the age of 15 years and whose time of HIV acquisition remained unknown, we estimated this time from the statistical model, based on their first CD4+ T-cell count upon arrival in France.

**Figure 1:**
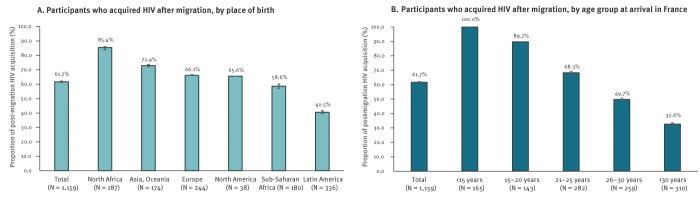
Proportions of participants who acquired HIV after migration, according to (A) place of birth (B) and age at arrival in France, Île-de-France, France, May 2021–June 2022 (n = 1,159 participants)

Overall, we estimated that a total of 715 participants (61.7%; 95% confidence interval (CI): 61.2–62.2)) had acquired HIV after arriving in France, while 444 participants (38.3%; 95%CI: 37.9–38.7) had acquired HIV before (Figure 1a). There was significant variability in rates of HIV acquisition after migration according to geographical area of origin; the rate of post-migration-HIV acquisition was highest among participants from North Africa (85.4%; 95%CI: 83.9–86.0) and was lowest among those from Latin America (40.5%; 95%CI: 39.6–41.6). These proportions also decreased with age at arrival (Figure 1b).

### Time between arrival in France and HIV acquisition

**Figure 2:**
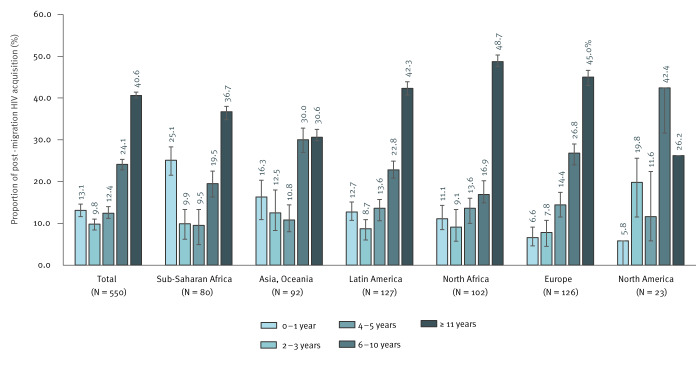
Time of HIV acquisition after migration, among participants who immigrated after the age of 15 years, according to place of birth, Île-de-France, France, May 2021–June 2022 (n = 550)

Among study participants who immigrated after the age of 15 years and acquired HIV after migration to France (n = 550), the median time from arrival to estimated date of HIV acquisition was 7.5 years (IQR: 3.5–14.75). Of these participants, we estimated that 13.1% (95%CI: 11.6–14.6) acquired HIV during their first year in France, and this proportion was higher during the first year than during the second and third years combined, for participants from sub-Saharan Africa, Asia and Oceania, Latin America and North Africa (Figure 2). Excess risk of HIV acquisition in the first year was particularly marked among participants from sub-Saharan Africa (25.1%; 95%CI: 21.5–28.3); this was not observed among participants from Europe and North America.

A total of 250 participants who were already aware of their HIV-positive status before migrating to France were identified. These individuals had been diagnosed for a median of 4.2 years (IQR: 2.1–8.0) before migrating to France. Among this group, 221 individuals (88.4%) had initiated ART before arriving in France, but 36/221 (16.3%) discontinued ART by the time they received medical care in France. In other words, of the 250 participants aware of their HIV-positive status before coming to France, 65 (26.0%) had either not started or had stopped ART upon arrival.

Among study participants who immigrated after the age of 15 years and acquired HIV after migration to France, 83/550 (15.1%) had a CD4+ T-cell count < 200/mm^3^, including 24/550 (4.4%) who had an acquired immunodeficiency syndrome (AIDS) defining event at entry into care. Median delay between estimated HIV acquisition time and first HIV medical visit was 2.0 years (IQR: 0.3–2.8).

### Reasons for migration and living condition at arrival in France

Among the 831 participants who arrived in France after the age of 15 years and who completed the questionnaire, the three most frequently given reasons for migration were: studying (309; 37.2%), sexual orientation (290; 34.9%), and experiencing a new country (261; 31.4%); medical necessity was mentioned as a reason for migration by only 84 participants (10.1%) (Table 2). In case of medical problems, HIV infection was reported as the main issue by 54 (64.3%) participants.

**Table 2:** Reasons for migration, social situation and sexual behaviour in the first 12 months after arrival in France, Île-de-France, France, May 2021–June 2022 (n = 831)

Situation	Number	Percentage
**Reasons for migration^a^**		
Studying	309	37.2
Sexual orientation	290	34.9
Discovering a new country	261	31.4
Escaping from insecurity or war	169	20.3
Economic considerations	177	21.3
Joining the partner	119	14.3
Escaping the family	107	12.9
Working	108	13
Medical issues	84	10.1
Joining the family	50	6
**Having felt forced to leave the country of birth**	453	54.5
**Social environment (people to rely on) at arrival^b^**		
Partner	132	15.9
Family	252	30.3
Friends	181	21.8
Colleagues	29	3.5
Non-governmental organisations	121	14.6
Healthcare workers	60	7.2
Nobody	257	30.9
**Practice of French language at arrival**		
Being able to speak	409	49.2
Being able to read	440	52.9
Being able to write	381	45.8
**Administrative/legal situation at arrival**		
No papers or seeking asylum	203	24.4
Temporary visa or residence permit	425	51.1
French^c^ or European Union citizenship	188	22.6
Missing	15	1.8
**Social insurance at arrival**		
None or state medical assistance^d^	231	27.8
Universal medical coverage^e^	227	27.3
Standard health coverage, including student health coverage	327	39.4
Missing	46	5.5
**Housing situation at arrival**		
Homeless	68	8.2
Hosted by family or friends	296	35.6
Owner or tenant of their own home	457	55
Missing	10	1.2
**Working situation at arrival**		
No work	227	27.3
Student^f^	241	29
Any paid work, including permanent and temporary contracts	353	42.5
Missing	10	1.2
**Subjective feeling on financial situation at arrival**		
Good	318	38.3
Insufficient	446	53.7
Very bad	46	5.5
Missing	21	2.5
**Number of sexual partners in the first 12 months**		
0	82	9.9
1	160	19.3
2–5	206	24.8
6–10	103	12.4
> 10	149	17.9
Missing	131	15.8
**Condomless sex in the first 12 months^g^**		
Yes, with all sexual partners	144	23.3
Only with occasional partners	76	12.3
Only with regular partners	65	10.5
With no one	332	53.7
Missing	1	0.2
**Means of meeting sexual partners in the first 12 months ^a,h^**		
Sexual meeting places (saunas, bars with darkroom)	150	32.8
Outside hook-up locations	90	19.7
Conviviality places (night clubs, bars)	202	44.2
Internet and connected apps	247	54

^a^ Participants could choose several reasons.

^b^ Participants could choose several reasons, excepting those who chose ‘Nobody’.

^c^ The definition of migrant used here is ‘born abroad’. People born abroad to French parents have French nationality by right and if they migrate to France later in life, they already have French nationality.

^d^ For people with irregular administrative/legal status.

^e^ For people with regular administrative/legal status and low incomes.

^f^ Students who reported a paid work were classified in the category ‘Any paid work’.

^g^ Among participants who reported having had at least one sexual partner in the last 12 months.

^h^ Among participants who reported having had at least two sexual partners in the last 12 months.

About half of study participants were not able to understand or speak French at arrival. A total of 203 participants (24.4%) were undocumented or were seeking asylum during the first 12 months on the French territory; 231 (27.8%) had no access to the French social insurance; 68 (8.2%) were homeless; 227 (27.3%) were unemployed and 492 (59.2%) felt they had insufficient income to support themselves. While 82 participants (9.9%) reported no sexual partners in the first 12 months in France, 149 (17.9%) had had more than 10, with inconsistent condom use. Of the 831 participants who arrived in France after the age of 15 years, 532 (64.0%) regularly visited their country of origin, and 212/532 (39.8%) reported having sexual intercourse upon their return in their country of origin.

### Factors associated with early HIV acquisition at arrival in France

We compared the demographic, socioeconomic characteristics, and sexual behaviours of participants who acquired HIV during the first year in France with those who acquired it later. In univariate analysis, participants who acquired HIV in the first year were older, more socially disadvantaged, and had more sexual partners than those who acquired HIV after the first year, as described in the Supplementary material, Table S1. In multivariable logistic regression analysis, participants who were older at arrival in France were more likely to acquire HIV within the first post-migration year (adjusted odds ratio (aOR): 1.12 per year; 95%CI: 1.06–1.18), as well as participants who came from sub-Saharan Africa (aOR: 9.90; 95%CI: 3.07–35.90) or Asia (aOR: 4.92; 95%CI: 1.59–16.66) in comparison with those who came from Europe, those who had more than 10 sexual partners during the first 12 months (aOR: 7.63 3.44–17.31), and those who were more disadvantaged, according to the social disadvantage indicator we built (aOR: 2.44; 95%CI: 1.17–5.05) (Table 3). This social disadvantage indicator included participants’ legal status, health coverage, employment, and financial well-being (see Methods and Supplementary material).

**Table 3:** Factors associated with the acquisition of HIV in the first 12 months after arrival, as assessed using binary logistic regressions, Île-de-France, France, May 2021–June 2022 (n = 403 participants with post-migration HIV acquisition and no missing data)

Characteristic		Univariate		Multivariate	
		OR	95%CI	aOR^a^	95%CI
**Age at arrival in France**	Per 1 year	1.10	1.05–1.15	1.12	1.06–1.18
**Place of birth**	Europe	Reference			
	Latin America	1.05	0.34–3.18	1.24	0.36–4.38
	North Africa	1.42	0.49–5.62	2.16	0.63–7.70
	Sub-Saharan Africa	4.49	1.76–12.53	9.90	3.07–35.90
	Asia, Oceania	2.79	1.06–7.92	4.92	1.59–16.66
	North America	0.90	0.05–5.62	0.83	0.04–5.78
**Having felt forced to leave the country of birth**	No	Reference			
	Yes	1.80	0.99–3.35	NA	NA
**Leaving the birth-country due to the sexual orientation**	No	Reference			
	Yes	2.47	1.35–4.52	NA	NA
**Leaving the birth-country due to health reasons**	No	Reference			
	Yes	3.58	0.16–30.06	NA	NA
**Social disadvantage indicator^b^**	< 9	Reference			
	≥ 9	3.38	1.82–6.26	2.44	1.17–5.05
**To be alone at the arrival in France**	No	Reference			
	Yes	0.72	0.38–1.44	NA	NA
**To speak French at the arrival in France**	Yes	Reference			
	No	1.29	0.71–2.34	NA	NA
**Number of sexual partners^c^**	≤ 10	Reference			
	> 10	4.80	2.46–9.24	7.63	3.44–17.31
**Use of condoms based on sexual partner^c^**	Yes, with all sexual partners	Reference			
	Only with occasional partners	0.39	0.06–1.64	NA	NA
	Only with regular partners	0.39	0.02–2.30	NA	NA
	With no one	1.89	0.85–4.65	NA	NA
	Not concerned^d^	0.69	0.26–1.90	NA	NA
**Meeting sexual partners in saunas, sex-clubs or outside hook-up locations^c^**	No	Reference			
	Yes	0.56	0.26–1.17	NA	NA
	Not concerned^e^	0.30	0.14–0.60	NA	NA
**Meeting sexual partners through Internet and dating apps^c^**	No	Reference			
	Yes	0.50	0.24–1.07	NA	NA
	Not concerned^e^	0.24	0.11–0.53	NA	NA

aOR: adjusted odds ratio; CI: confidence interval; NA: not applicable; OR: odds ratio.

^a^ Multivariate model included the age at arrival in France, the place of birth, the fact of leaving the birth-country due to the sexual orientation as a reason for migration, the social disadvantage indicator (participants' administrative/legal status, health coverage, employment situation, and financial well-being), and the number of sexual partners.

^b^ The social disadvantage indicator included the administrative/legal status, the working situation, the social insurance, and the subjective feeling on financial situation in the 12 months after arrival in France (see the Methods section of the article and the Supplementary material).

^c^ During the first year after arrival in France.

^d^ No sexual partners during the first year after arrival in France.

^e^ Less than two sexual partners during the first year after arrival in France.

## Discussion

In this study conducted in France, we found that 62% of migrant MSM acquired HIV after migrating to the country. This high proportion is consistent with previous estimations ranging from 39 to 72%, which have been obtained in other European countries since the 2010s [5,7,19,20], and is higher than in heterosexual migrant populations. The places of origin of our study population, representative of MSM living with HIV currently in HIV care in the Paris region, clearly differed from heterosexuals living with HIV in France, who are overwhelmingly from sub-Saharan Africa [15]. Moreover, the diversity in such places highlights the heterogeneity of this MSM population with a considerable proportion coming from Latin America and Europe, and the remainder evenly distributed between North Africa, sub-Saharan Africa and Asia. We showed notable variations in the proportion of people who acquired HIV after migration, depending on participants’ origins (ranging from 41% for Latin America to 85% for North Africa). In addition, we showed that the lower the age at migration to France, the higher the probability of acquiring HIV after migration, as suggested elsewhere in Europe between 2007 and 2016 [19]. A large proportion of North African participants migrated to France at a very young age, due to the historical links between the Maghreb and France, which may partly explain the high rate of post-migration HIV acquisition in this sub-population. However, we hypothesise that contextual factors (e.g. legal status of homosexuality and economic status in the country of origin) and individual factors (e.g. reason for migration and sexual behaviour) primarily determine the risk of HIV acquisition before, during and after the migration process. Finally, it is important to note that acquiring HIV after migration does not necessarily mean acquiring HIV in France; indeed, we know that two-thirds of the participants returned regularly to their country of birth, and that around 40% of them also engaged in sexual intercourse there.

Among participants who acquired HIV after migration, we estimated that 13% acquired HIV within the first year; this proportion reached 25% in participants who came from sub-Saharan Africa. To our knowledge, our study is the first to suggest an increased risk of contracting HIV early after migration among MSM. This finding should shape public health and prevention policies and highlights the need to investigate the causes of this excess risk.

We collected information on the social and economic situation during the first year in France and found a high degree of social disadvantage in the study population. Indeed, almost half of the participants did not speak French on arrival; 24% were undocumented; 28% had no access to French social insurance; 8% were homeless; 27% were unemployed; and more than half felt they had insufficient income to support themselves. In the past decade, in Europe, several studies have identified irregular status [21], economic disadvantage [22], administrative complexity [23], or understanding and communication problems [24,25] as obstacles to HIV risk management (including HIV testing) among migrants [26,27], particularly in the first years after migration. It should be noted that most studies have focused on heterosexual populations and very few concern migrant MSM [28]. We believe that the high degree of social disadvantage of the participants in the GANYMEDE study made them vulnerable to HIV, notably due to a lack of access to the healthcare system and prevention services. Although HIV pre-exposure prophylaxis (PrEP) was not yet approved at the time of migration for most participants, it is likely that MSM experiencing precarity and who have recently migrated miss out on opportunities to benefit from PrEP. Currently, a number of community initiatives are informing newly arriving migrants about PrEP and facilitating their access to it. We believe that simplified circuits should be created between these field actions and specialised care settings. However, as previously described, our study population was very heterogeneous and also included a considerable proportion of participants with a relatively high social and economic status (55% with their own home, and 43% with a legal employment contract), who likely did not experience these specific barriers.

HIV transmission risk cannot be understood without studying the sexual behaviour of migrant MSM. In our study 65% of migrant MSM with relevant information available had had more than one sexual partner, including 21% with more than 10 partners, within the first year following their arrival in France. Further, 77% used condoms inconsistently or not at all within the first 12 months in France. As reported in a review published in 2017, previous studies have shown a high prevalence of condomless sex, including during periods without access to PrEP, associated with a high prevalence of drug and alcohol use among migrant MSM in Europe and North America [29]. There is also evidence of sexual mixing between established and migrant populations based on virus phylogenetic analyses and survey results [5]. We hypothesise that sexual encounters both within migrant communities and in new sexual networks may lead to HIV acquisition, especially in the context of insufficient access to prevention. In addition, an American qualitative study conducted in 2015 has demonstrated the complexity and risks of entering a new sexual scene in a new country, particularly in large cities [11]. In our study, sexual orientation was highlighted as a reason for migration by a third of participants. We believe that radical changes in the sexual behaviours of participants from countries where homosexuality is repressed may have led to increased exposure to HIV. This hypothesis is supported by a study conducted in 2019 among Latin American migrant MSM living in New York City, where escaping violence or persecution associated with homosexuality was strongly associated with post-migration HIV acquisition [30]. In parallel, only 6% (54/831) of participants indicated that they had left their country of birth due to HIV-related medical problems; this refutes the common belief of ‘medical tourism’ associated with migrant PLWH, i.e. going to another country for treatment.

By cross-referencing the estimated time of HIV acquisition after migrating to France with demographic, social and sexual determinants, we were able to determine predictors of early HIV acquisition in France. An older age at arrival, certain places of origin (sub-Saharan Africa and Asia), a higher level of social disadvantage and a greater number of sexual partners were independently associated with acquiring HIV after migration during the first year in France. These predictors are of great importance when it comes to designing preventive actions, both for associations working locally with newly arriving MSM migrants and for health authorities defining national prevention strategies.

Our study has some limitations. We only included participants who disclosed having had sex with other men, as they were identified first with the self-reported route of HIV transmission; we cannot exclude that participants who were uncomfortable with their homosexuality would have come from specific places and would have had different sexual behaviour and levels of social disadvantage. All participants included in the study were engaged in HIV care, and by definition, this excludes MSM migrants living with HIV without knowing it or refusing care. Another factor is potential memory bias, since half the participants arrived in France more than 15 years ago, which may have affected the accuracy of some responses. Finally, the quantitative nature of the study prevented us from capturing the diversity of individual life stories. A qualitative approach would complement our results, for example to explore the complex ways in which socioeconomic and sexual vulnerabilities are intertwined on arrival in France.

### Conclusion

In conclusion, we show that a high proportion of migrant MSM living in the Paris region acquire HIV after migrating to France, and that a considerable proportion of them acquire it in the first few years after arrival. We believe that this increased risk can be explained by the accumulation of vulnerability factors and changes in sexual behaviour, particularly as a result of a more permissive environment to have sex with other men.

The results of this research work may be helpful to guide prevention policies in France, and to facilitate engagement with the most vulnerable migrants arriving in the country while responding to the factors that contribute to their vulnerability. Moreover, it is critical that the rate of post-migration HIV acquisition is continuously monitored to assess and adapt prevention policies accordingly; the two-pronged method based on simple biographical data (age of first sexual intercourse, last negative HIV test) and biological data (first available CD4+ T lymphocyte count, evidence of primary infection) that we used in the GANYMEDE study should be implemented routinely.

## Supplementary Data

1141000 Supplementary Material
