# Supplementary material for: High proportion of post-migration HIV acquisition in migrant men who have sex with men receiving HIV care in the Paris region, and associations with social disadvantage and sexual behaviours: results of the ANRS-MIE GANYMEDE study, France, 2021 to 2022
Source: Euro Surveill. 2024 Mar 14;29(11):2300445. doi: 10.2807/1560-7917.ES.2024.29.11.2300445 (PMC10941311; doi:10.2807/1560-7917.ES.2024.29.11.2300445)
Supplement: Supplementary Material [file 2300445_SupplementaryMaterial.pdf]

This supplementary material is hosted by *Eurosurveillance* as supporting information alongside the article "High proportion of post-migration HIV acquisition in migrant men who have sex with men receiving HIV care in the Paris region, and associations with social disadvantage and sexual behaviours: results of the ANRS-MIE GANYMEDE study, France, 2021 to 2022,", on behalf of the authors, who remain responsible for the accuracy and appropriateness of the content. The same standards for ethics, copyright, attributions and permissions as for the article apply. Supplements are not edited by *Eurosurveillance* and the journal is not responsible for the maintenance of any links or email addresses provided therein.

Supplementary File

1. Figure S1. Geographic distribution of treatment centres associated to the ANRS-MIE GANYMEDE study (p. 2)

2. Figure S2. Classification of timing of HIV acquisition according to questionnaire and medical records, before using statistical model (p. 3)

3. Table S1. Distribution of sexual and socio-economic variables by timing of HIV acquisition among migrant MSM who acquired HIV in France (p. 4)

4. Supplementary details on the seroconversion model (p. 6)

5. Details on the study of factors associated with early HIV acquisition after arrival in France (p. 13)

6. References (p. 19)

Figure S1. Geographic distribution of treatment centres associated to the ANRS-MIE GANYMEDE study.

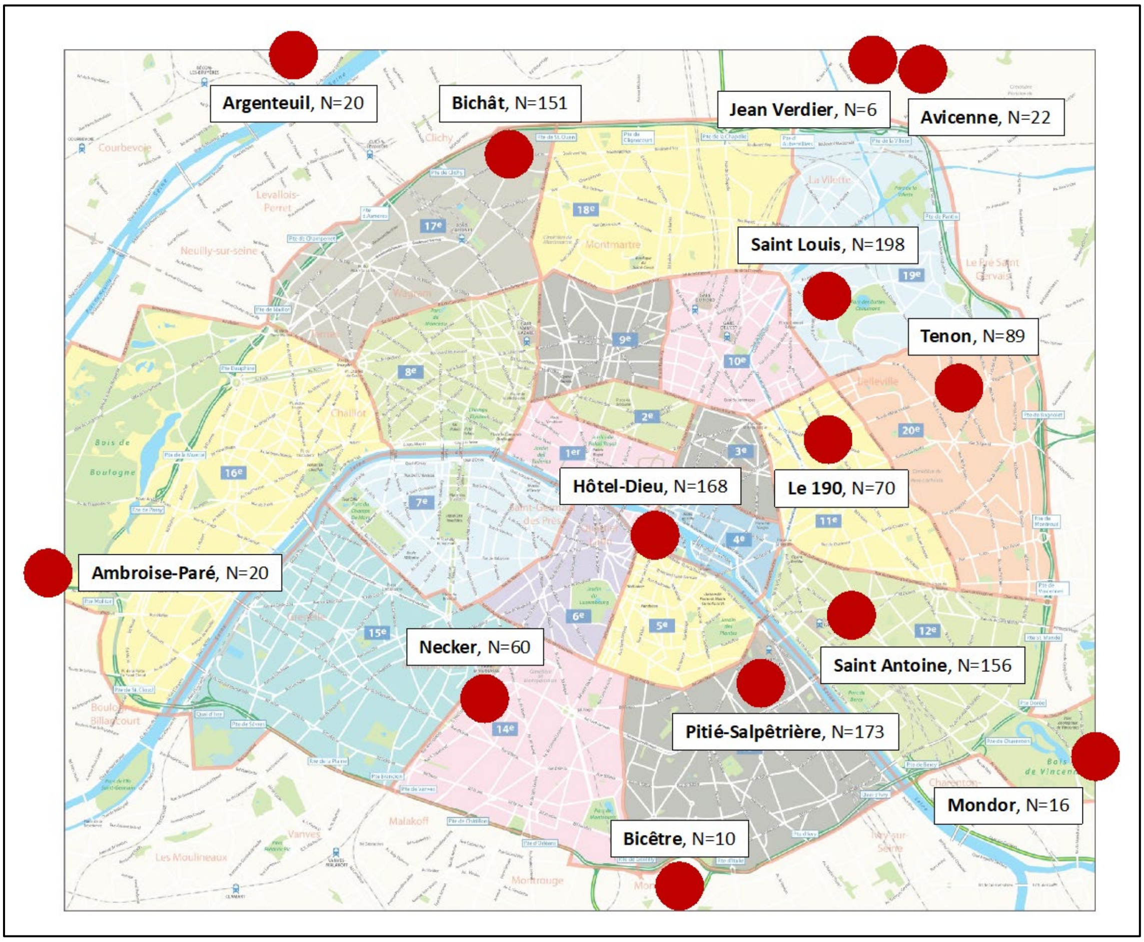

Figure S2. Classification of timing of HIV acquisition according to questionnaire and medical records, before using statistical model.

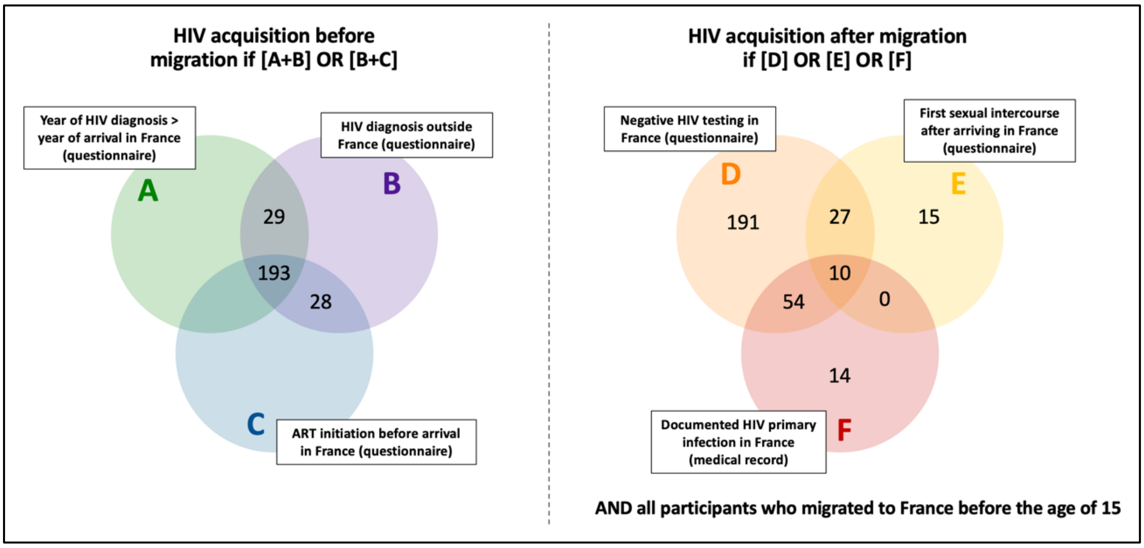

Table S1. Distribution of sexual and socio-economic variables by timing of HIV acquisition among migrant MSM who acquired HIV in France (N=403[1]).

	Total (N=403)	HIV acquisition within the first year in France (N=50)	HIV acquisition after the first year in France (N=353)
Age at arrival in France, years (median, IQR)	24 (21-29)	28 (24-35)	24 (20-28)
Region of birth			
Europe	95 (23.6%)	7 (14.0%)	88 (24.9%)
Latin America and the Caribbean	91 (22.6%)	7 (14.0%)	84 (23.8%)
North Africa	79 (19.6%)	8 (16.0%)	71 (20.1%)
Sub-Saharan Africa	57 (14.1%)	15 (30.0%)	42 (11.9%)
Asia – Oceania	66 (16.4%)	12 (24.0%)	54 (15.3%)
North America	15 (3.7%)	1 (2.0%)	14 (4.0%)
Social disadvantage indicator			
<9	309 (76.7%)	27 (54.0%)	282 (79.9%)
≥9	94 (23.3%)	23 (46.0%)	71 (20.1%)
Number of sexual partners during the first 12 months in France			
<=10	344 (85.4%)	31 (62.0%)	313 (88.7%)
>10	59 (14.6%)	19 (38.0%)	40 (11.3%)
Administrative status during the first 12 months in France			
French or European nationality	88 (21.8%)	6 (12.0%)	82 (23.2%)
Visa / residency permit	230 (57.1%)	26 (52.0%)	204 (57.8%)
Irregular status / asylum request	85 (21.1%)	18 (36.0%)	67 (19.0%)
Working situation during the first 12 months in France			
Permanent or temporary contract	163 (40.4%)	18 (36.0%)	145 (41.1%)
Student	153 (38.0%)	11 (22.0%)	142 (40.2%)
Unemployed / irregular job	87 (21.6%)	21 (42.0%)	66 (18.7%)
Health coverage during the first 12 months in France			
General regime of social security	218 (54.1%)	17 (34.0%)	201 (56.9 %)
CMU / CSS	98 (24.3%)	14 (28.0%)	84 (23.8%)
AME or nothing	87 (21.6%)	19 (38.0%)	68 (19.3%)
Financial situation during the first 12 months in France			
Comfortable	182 (45.2%)	15 (30.0%)	167 (47.3%)
Tight or struggle to make ends meet	204 (50.6%)	28 (56.0%)	176 (49.9%)
Impossible to make ends meet	17 (4.2%)	7 (14.0%)	10 (2.8%)
Housing situation during the first 12 months			
Tenant, co-tenant or owner	232 (57.6%)	27 (54.0%)	205 (58.1%)
Housed by friends or family	142 (35.2%)	19 (38.0%)	123 (34.8%)
Housed by an association / homeless	29 (7.2%)	4 (8.0%)	25 (7.1%)
Having felt forced to leave the country of birth			
Not at all	204 (50.6%)	19 (38.0%)	185 (52.4%)
Somewhat or quite compelled to leave	199 (49.4%)	31 (62.0%)	168 (47.6%)
Leaving the birth-country due to the sexual orientation			
No	283 (70.2%)	26 (52.0%)	257 (72.8%)
Yes	120 (29.8%)	24 (48.0%)	96 (27.2%)
Leaving the birth-country due to health reasons			
No	400 (99.3%)	49 (98.0%)	351 (99.4 %)
Yes	3 (0.7%)	1 (2.0%)	2 (0.6 %)
To be alone at the arrival in France			
No	91 (22.6%)	14 (28.0%)	77 (21.8%)
Yes	312 (77.4%)	36 (72.0%)	276 (78.2%)
To speak French at the arrival in France			
Yes	224 (55.6%)	25 (50.0%)	199 (56.4%)
No	179 (44.4%)	25 (50.0%)	154 (43.6%)

(continued)	Total (N=403)	HIV acquisition within the first year in France (N=50)	HIV acquisition after the first year in France (N=353)
Use of condoms during the first 12 months in France			
Always	70 (17.4%)	8 (16.0%)	62 (17.6%)
Not always	211 (52.4%)	32 (64.0%)	179 (50.7%)
Not concerned[2]	122 (30.3%)	10 (20.0%)	112 (31.7%)
Use of condoms based on sexual partner during the first 12 months in France			
Yes, with all sexual partners	70 (17.4%)	8 (16.0%)	62 (17.6%)
Only with occasional partners	42 (10.4%)	2 (4.0%)	40 (11.3%)
Only with regular partners	21(5.2%)	1 (2.0%)	20 (5.7%)
With no one	148 (36.7%)	29 (58.0%)	119 (33.7%)
Not concerned[2]	122 (30.3%)	10 (20.0%)	112 (31.7%)
Meeting sexual partners in saunas, sex-clubs or outside hookup locations			
No	116 (28.8%)	24 (48.0%)	92 (26.1%)
Yes	94 (23.3%)	12 (24.0%)	82 (23.2%)
Not concerned (less than 2 sexual partners)	193 (47.9%)	14 (28.0%)	179 (50.7%)
Meeting sexual partners through internet and dating apps			
No	61 (15.1%)	15 (30.0%)	46 (13.0%)
Yes	149 (37.0%)	21 (42.0%)	128 (36.3%)
Not concerned (less than 2 sexual partners)	193 (47.9%)	14 (28.0%)	179 (50.7%)
Meeting sexual partners in saunas, sex-clubs or outside hookup locations or through internet and dating apps			
No	66 (16.4%)	9 (18.0%)	57 (16.1%)
Yes	144 (35.7%)	27 (54.0%)	117 (33.1%)
Not concerned (less than 2 sexual partners)	193 (47.9%)	14 (28.0%)	179 (50.7%)

1. Among the 449 individuals who acquired HIV in France, all the analysis were performed based on 403 individuals with complete information on the explanatory variables of the model. 2. No sexual partners during the first year after arrival in France.

Supplementary details on the seroconversion model

A seroconversion model utilizing CD4 count data was used to estimate the time of HIV acquisition for participants who arrived in France at or after the age of 15 but had an unknown timing of HIV acquisition.

While various CD4-stage models exist,[1-3] predominantly based on Markovian frameworks, difficulties arose when fitting these models to accurately estimate seroconversion timing. To overcome these challenges, a non-Markovian model called *"stochastic chains with memory of variable length"* was utilized. This approach, also known as *Variable Length Markov Chains* (VLMC), was introduced by Rissanen[4] and further elaborated by Bühlmann and Wyner.[5] VLMCs have been successfully applied in diverse domains such as DNA sequence analysis,[5] protein classification[6] and linguistic data analysis.[7] Below, we briefly explain what these types of models consist of, and then we will show their application to the GANYMEDE study.

In a Markov chain of order *k*, the relevant portion of the past which influences the next outcome in a sequence is always the last k observations. This means that:

$$\begin{aligned} P(X_t = x_t \mid X_{t-1} = x_{t-1}, \dots, X_1 = x_1) \\ = P(X_t = x_t \mid X_{t-1} = x_{t-1}, \dots, X_1 = x_{t-k}) \\ = P(X_t = x_t \mid X_{t-k}^{t-1} = x_{t-k}^{t-1}) \end{aligned} \quad (1)$$

In the expressions (1), the standard notation is used, where random variables are denoted by uppercase letters and their observed values by lowercase letters. Furthermore, the sequence of length $j - i + 1$, $(x_i, x_{i+1}, \dots, x_j)$, is denoted by x_i^j.

In contrast to Markov chains, the portion of the past referred to as the context in stochastic chains with variable length memory is not always constant. The context is determined by a function c, which takes the entire past as input and returns a sequence of length *l*, consisting of the last *l* observations of the chain. The value of *l* is defined as:

$$l = \min\{k, P(X_t = x_t \mid X_1^{t-1} = x_1^{t-1}) = P(X_t = x_t \mid X_{t-k}^{t-1} = x_{t-k}^{t-1}) \quad \forall x_t\} \quad (2)$$

One of the key tasks when utilizing stochastic chains with variable length memory is to identify the function c, in order to determine the set of contexts. A special property of the function c is that if the string $x_j, x_{j-1}, \dots, x_1$ is deemed a context, then none of its sub-strings $x_k, x_{k-1}, \dots, x_1$ with $1 \leq k \leq j - 1$ are also recognized as contexts. Thanks to this property, the contexts can be represented by a tree with a root node at the top, and branches growing backwards.

As an example, let's suppose a binary random variable X which can take the values 0 and 1. Let's also consider that this variable is observed over time, and we observe the sequence $x_1, x_2, \dots, x_T$. Assuming that this sequence is governed by a variable-length memory stochastic chain, whose context function is defined by:

$$c(x_{-\infty}^{-1}) = \begin{cases} 0, & \text{if } x_{-1} = 0 \\ 1,1, & \text{if } x_{-1} = 1,\ x_{-2} = 1 \\ 0,0,1, & \text{if } x_{-1} = 1,\ x_{-2} = 0,\ x_{-3} = 0 \\ 1,0,1, & \text{if } x_{-1} = 1,\ x_{-2} = 0,\ x_{-3} = 1 \end{cases}$$

So, that context function can be represented by the following tree:

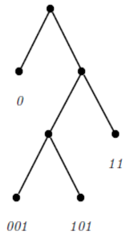

This means that each context is represented as a branch (or terminal node) of the tree. The context of length *l*, $w = x_l^1$ is represented by a branch, whose subbranch in the top indicate that in order to predict the state at the time t given the whole past is given by:

$$\begin{aligned} P(X_t = x_t | X_{t-1} = x_{t-1}, X_{t-2} = x_{t-2}, \dots, X_1 = x_1) \\ = P\big(X_t = x_t \big| c(x_{t-1}, x_{t-2}, \dots x_1)\big) \\ = P(X_t = x_t | x_{t-1}, x_{t-2}, \dots x_{t-l}) \end{aligned} \quad (3)$$

where $c(x_{t-1}, x_{t-2}, \dots x_1) = (x_{t-1}, x_{t-2}, \dots, x_{t-l})$ represents the portion of the past which is necessary to predict the state of the chain at time t. This means that each context is associated with a set of transition probabilities that allow us to predict the state of the chain at time t, immediately after the occurrence of that context.

The combination of all contexts and their associated transition probabilities defines what we call the *probabilistic context tree*, which constitutes the stochastic model associated with the observed sample. Given an observed sample $x_1, x_2, \dots, x_T$, we can estimate its underlying probabilistic context tree thanks to the context algorithm which will be explained below.

The context algorithm:

Given a random variable X taking values in a finite space A, and assuming that X is observed along the time. Given the sample $x_1, x_2, \dots, x_T$, then the estimation algorithm works as follows: First, a maximal tree is selected that includes all branches that appear a minimum number of times in the sample. Subsequently, the branches of this tree are pruned systematically until the smallest tree is obtained, which best fits the data. The decision to prune a context is determined by evaluating a specific gain function, such as the log-likelihood ratio.

Consequently, the estimated context tree is the largest tree such that:

$$\Delta_{wu} = \sum_{x \in A} \left\{ \hat{P}(x|wu) \log\left(\frac{\hat{P}(x|wu)}{\hat{P}(x|w)}\right) N(wu) \right\} \geq K$$

With $K = K_n \to \infty (n \to \infty)$ and $\hat{P}(\cdot \mid \cdot)$ defined by:

$$\hat{P}(x|w) = \frac{N(xw)}{N(w)}$$

Such that $x, w \in \bigcup_{m=1}^{\infty} A^m$, $xw = (\dots, x_2, x_1, \dots, w_2, w_1)$, and

$N(w) = \sum_{t=1}^{n} I_{\{X_t^{t+|w|-1} = w\}}$, this is the number of occurrences of the sequence w in the observed sample.

Selection of the context model in seroconversion data:

Data from the French Hospital Database on HIV (ANRS CO4 FHDH) was employed, which is a large hospital-based study ongoing since 1989 with 22.756 patients in France, providing information on HIV seroconversion and CD4 T-cell counts prior to ART initiation.

In our study, the state space of the stochastic chain represents various CD4 cell levels in individuals. We have defined a state space comprising six states, each corresponding to a specific CD4 cell count range: state 1 for counts >500 cells/mm3, state 2 for counts between 350-500 cells/mm3, state 3 for counts between 200-350 cells/mm3, and state 4 for counts <200 cells/mm3. Additionally, we have included state 0 to symbolize seroconversion, the transition from HIV-negative to HIV-positive status, and state 9 to indicate situations where information about the CD4 count is unavailable. The model assumes a retrospective increase in CD4 count until reaching the "Seroconversion state".

For each individual in the FHDH cohort, we established a quarterly state among the six mentioned above. In this way, each individual has a sequence of CD4 cell levels before initiating treatment. The length of these sequences varies according to the available information for each patient. The following table displays the distribution of the maximum lengths of the CD4 level sequences.

Maximum length observed	1	2	3	4	5	6	7	8	9	≥10	Total
Freq.	5827	2213	1182	770	467	287	205	127	89	241	11408
%	51,1	19,4	10,4	6,7	4,1	2,5	1,8	1,1	0,8	2,1	100,0

We explored a total of 25 potential models to predict the quarter of seroconversion for individuals in the FHDH cohort. These models ranged from simple first-order Markov chain to more complex ones involving context trees with a height of 4.

Using these potential models and the latest CD4 cell measurement prior to initiating HIV treatment, we identified the seroconversion quarter by generating 50 simulations of the CD4 state sequence until finding the quarter corresponding to the patient's seroconversion. For each simulation, we calculated the time gap between the last CD4

measurement before treatment initiation and the moment of seroconversion for each individual. Through calculating the mean of these values, we identified the potential quarter in which the individuals likely acquired HIV, assuming that the underlying model for simulation was appropriate.

This approach allowed us to compare observed and simulated seroconversion quarters and evaluate the accuracy of our predictions for each model. Among all the explored models, the one with the best fit had the following context tree structure:

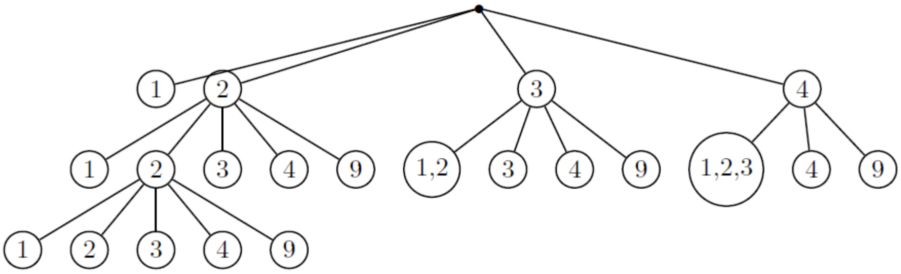

Furthermore, the transition probabilities associated to each context are as follows:

Context c	$p(0\|c)$	$p(1\|c)$	$p(2\|c)$	$p(3\|c)$	$p(4\|c)$
c(1)	0.1259	0.7220	0.1403	0.0108	0.0011
c(9,2)	0.1049	0.2674	0.5175	0.1087	0.0014
c(9,3)	0.1133	0.0526	0.3474	0.4661	0.0205
c(9,4)	0.2022	0.0127	0.0526	0.3028	0.4297
c(1,2)	0.1074	0.4751	0.3874	0.0288	0.0014
c(3,2)	0.0464	0.1429	0.5921	0.2164	0.0021
c(4,2)	0.0833	0.3333	0.4583	0.0833	0.0417
c(9,2,2)	0.0576	0.2673	0.5819	0.0927	0.0005
c(1,2,2)	0.0399	0.3789	0.5242	0.0570	0.0000
c(2,2,2)	0.0463	0.2774	0.6057	0.0698	0.0007

Context c	$p(0\|c)$	$p(1\|c)$	$p(2\|c)$	$p(3\|c)$	$p(4\|c)$
c(3,2,2)	0.0519	0.1851	0.5813	0.1817	0.0000
c(4,2,2)	0.0000	0.2000	0.4000	0.4000	0.0000
c(1,3)	0.0817	0.1272	0.5099	0.2730	0.0082
c(2,3)	0.0817	0.1272	0.5099	0.2730	0.0082
c(3,3)	0.0615	0.0364	0.3764	0.5106	0.0151
c(4,3)	0.0632	0.0287	0.2644	0.5747	0.0690
c(1,4)	0.1806	0.0556	0.1528	0.4722	0.1389
c(2,4)	0.1806	0.0556	0.1528	0.4722	0.1389
c(3,4)	0.1806	0.0556	0.1528	0.4722	0.1389
c(4,4)	0.1657	0.0118	0.0473	0.3787	0.3964

From these results, we can conclude that individuals with higher CD4 counts, such as above 500 cells/mm3, have a higher probability of seroconversion in the immediately preceding quarter, with probabilities ranging from 0.1657 to 0.2022. As the CD4 count decreases within the range of 350 to 500 cells/mm3, the probability of seroconversion decreases and fluctuates between 0.0632 and 0.1133. Similarly, for individuals with CD4 count between 200 and 350 cells/mm3, the probability of seroconversion in the precedent quarter oscillates between 0 and 0.1049. Finally, if we observe a CD4 cell count below 200, the estimated probability of seroconversion is 0.1259.

Validation of the model:

Initially, to validate the model, we compared the observed and simulated average time intervals between seroconversion and the last recorded CD4 count before treatment initiation. The observed average delay in the FHDH cohort was found to be 9.89 quarters (equivalent to 2.47 years), while the model's simulations produced an average delay of 9.91 quarters (also 2.47 years).

The mean difference between the observed and simulated delays was 0.107 whose 95% confidence interval is (-0.107, 0.320), suggesting that the difference is not statistically

significant. These results provide evidence of the model's ability to accurately estimate the time interval between seroconversion and the last CD4 count, reinforcing its validity in predicting seroconversion times.

As a second approach validation, we consider the information for the individuals in the GANYMEDE study with a known seroconversion timing (this is before or after the arrival in France). The validation results of the model using data from the GANYMEDE study are as follows:

In the GANYMEDE study, there were 506 individuals aged over 15 years with known seroconversion timing. The model correctly identified the seroconversion timing in 83.5% of these individuals, with a 95% confidence interval of 81.2% to 85.6%.

For the 158 individuals in the study who were younger than 15 years and had known seroconversion timing, the model achieved an impressive accuracy rate. It correctly identified the seroconversion timing in 99.9% of these individuals, with a 95% confidence interval of 99.4% to 100.0%.

When considering all individuals in the GANYMEDE study with known seroconversion timing, which includes both those aged over 15 years and those younger than 15 years, the overall proportion of individuals for whom the model accurately predicted the seroconversion timing was 87.4%, with a 95% confidence interval of 85.7% to 89.0%. This indicates that the model performed well in accurately identifying the seroconversion timing for the majority of individuals across different age groups in the study.

These validation results provide evidence of the model's effectiveness in predicting the seroconversion timing in the GANYMEDE study population. The high accuracy rates observed in both the older and younger age groups demonstrate the reliability and usefulness of the model in accurately predicting seroconversion timing for individuals in different age categories.

Likely timing of HIV acquisition:

In the GANYMEDE study, the likely timing of HIV acquisition is defined as a binary variable indicating whether the acquisition occurred after or before the arrival in France. The initial step in determining the likely timing of HIV acquisition involved utilizing data from the questionnaire and medical records, according to the following criteria:

1. For participants who arrived in France after the age of 15, it was presumed that they acquired HIV before migrating if their questionnaire indicated that they became aware of their positive HIV status in a country other than France. This assumption was further supported if they reported in their questionnaire a year of HIV diagnosis prior to their arrival in France or a year of ART initiation prior to their arrival in France.
2. Participants who did not meet the aforementioned criteria were classified as having acquired HIV post-migration if they reported any of the following in their self-questionnaire or medical records: (i) their first sexual intercourse taking place

in France (self-questionnaire), (ii) a negative HIV test in France (self-questionnaire), or (iii) a diagnosis of primary infection at least one year after their arrival in France (medical records).
3. If none of these criteria were met, the timing of HIV acquisition was considered unknown.
4. Lastly, for individuals who arrived in France before the age of 15, it was assumed that they acquired HIV after their migration.

(See Figure S2, above.)

Out of the 831 participants who completed the questionnaire, we were able to determine the likely timing of HIV acquisition for 559 individuals (67.3%), using data from questionnaires and medical records. However, for the remaining 272 individuals (32.7%) with unknown timing of HIV acquisition, we utilized the seroconversion model. We estimated their likely timing of HIV acquisition by considering their first CD4 cell count upon arrival in France.

One of the objectives of the GANYMEDE study is to identify factors associated with early HIV acquisition after arrival in France. In order to achieve this, we implemented an algorithm to estimate the quarter of seroconversion for individuals who were previously classified as acquiring HIV after their arrival in France. The algorithm follows these steps:

1. Based on data collected from questionnaires and medical records, we initially classify the probable timing of HIV acquisition into three categories: Before arrival in France, after arrival in France, and unknown. This classification is referred to as "Classification Q."
2. Using the seroconversion model, which considers the individual's initial CD4 cell count and the corresponding date, we simulate the estimated quarter of HIV acquisition, denoted as Q.S.M. Subsequently, we compare this quarter with the quarter of arrival in France to derive a second classification known as "Classification M." This classification categorizes the probable timing of HIV acquisition as occurring either before or after the individual's arrival in France.
3. For individuals who have evidence of a primary infection diagnosed in France based on medical records, we consider the quarter of seroconversion to be the same as the quarter of diagnosis in France.
4. By comparing the results from Classification Q and Classification M, we determined the probable quarter of seroconversion for individuals who acquired HIV after arriving in France, based on the following rules:
    a. If both Classification Q and Classification M indicate that the acquisition occurred after arrival in France, the probable seroconversion quarter is set as Q.S.M, which corresponds to the quarter simulated by the seroconversion model.
    b. If Classification Q indicates that the acquisition occurred after arrival in France, but Classification M indicates that the virus acquisition happened before arrival in France, we consider that the individual indeed acquired HIV after arrival in France. The probable seroconversion quarter is then

set as the midpoint between Q.S.M and the quarter when the first CD4 cell count was obtained.

c. If Classification Q indicates that the timing of HIV acquisition is unknown, we use Classification M, and Q.S.M is considered as the probable seroconversion quarter.

Details on the study of factors associated with early HIV acquisition after arrival in France

From this point onwards, the analysis focuses on individuals who were classified in the category of HIV acquisition after arrival in France. In the Table S1 *(see above)*, we show a comparison between participants who acquired HIV during the first year after arrival in France and those who acquired it after the first year, as a part of the descriptive analysis that we conducted in the preliminary stages of the identification of factors associated with early HIV acquisition after the arrival in France.

To investigate the risk factors associated with HIV acquisition within the first year after arrival in France among individuals who acquired HIV after migration, a series of analyses were performed. The goal was to identify the explanatory variables to be included in a multivariate logistic regression model.

In order to identify identify associations between the levels of different categorical variables, we performed several Multiple Correspondence Analysis (MCA). The correspondence analysis is a descriptive technique which aims to identify associations between the categories of different categorical variables. The analysis is based on a contingency table, where each cell of the table contains the number of observations that have a particular combination of levels of the variables. The goal of the analysis is to represent these data in a low-dimensional space, so that it is possible to visualize the patterns of association between the variables.

In an MCA plot, we can determine the associations between categories by observing their proximity and clustering on the plot. Several key aspects should be considered when interpreting an MCA plot:

1. Categories that are located close to each other on the MCA plot indicate a higher level of association. The closer the categories are, the more similar their responses or characteristics.
2. Groups or clusters of categories that are tightly grouped together on the MCA plot suggest associations between the categories within them.
3. Categories located in similar directions or aligned along an axis are more likely to be associated. Conversely, categories that are positioned in opposite directions or on different axes are less likely to be associated.

Social disadvantage indicator:

The first step involved conducting univariate logistic regressions to identify potential risk factors associated with HIV acquisition. The GANYMEDE study included several questions related to patients' situation during the first 12 months after arrival in France. These variables included participants' administrative status, health coverage, employment situation, and financial well-being.

To prepare for the subsequent multivariate analyses and to address any potential issues of multicollinearity, an indicator of social disadvantage was constructed using the

aforementioned variables. Each variable was transformed into an ordinal variable with three levels, where level 3 represented the most precarious conditions such as irregular administrative status, lack of medical coverage, unemployment or irregular employment, and insufficient economic resources. On the other hand, level 1 represented the best conditions, encompassing factors like French or European nationality, regular medical coverage, and consistent employment with sufficient economic resources. The social disadvantage indicator was derived by summing the contributions from each variable, resulting in a range of scores from 4 to 12. Using the cumulative square root frequency method, two groups were identified. Individuals with an indicator score equal to or greater than 9 were classified as being in a precarious situation.
Initially, we used the MCA in order to describe the social disadvantage indocator (CAT_INDEX_AR2), based on its four underlying variables: administrative status (ADM_STATUS), health coverage (HEALTH_COVR), employment situation (WORK_SIT), and financial well-being (FINANCE_AR). The MCA plot is shown below.

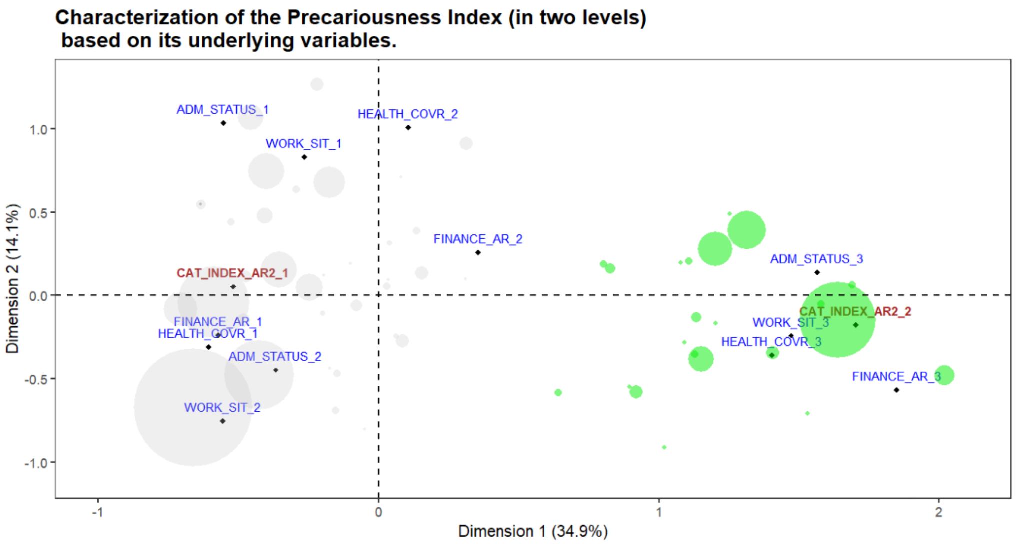

In the correspondence analysis plot, individuals belonging to category 2 of the social disadvantage indocator are represented in green, while those in category 1 are represented in grey. It is evident that this binary classification of the indicator strongly differentiates individuals who acquired the virus after arriving in France.

This analysis suggests that the second level of the social disadvantage indocator is associated with individuals who experience the most vulnerable situation during their first year in France. Specifically, they are characterized by unemployment, lack of health coverage, irregular immigration status or pending asylum requests, and financial difficulties.

A second analysis was conducted, this time including not only the underlying variables of the social disadvantage indocator but also the response variable indicating whether the acquisition of the virus occurred early during the first year after arrival in France (TimeHIVacq1_1) or if it occurred after the first year in France (TimeHIVacq1_0). The MCA plot is shown below.

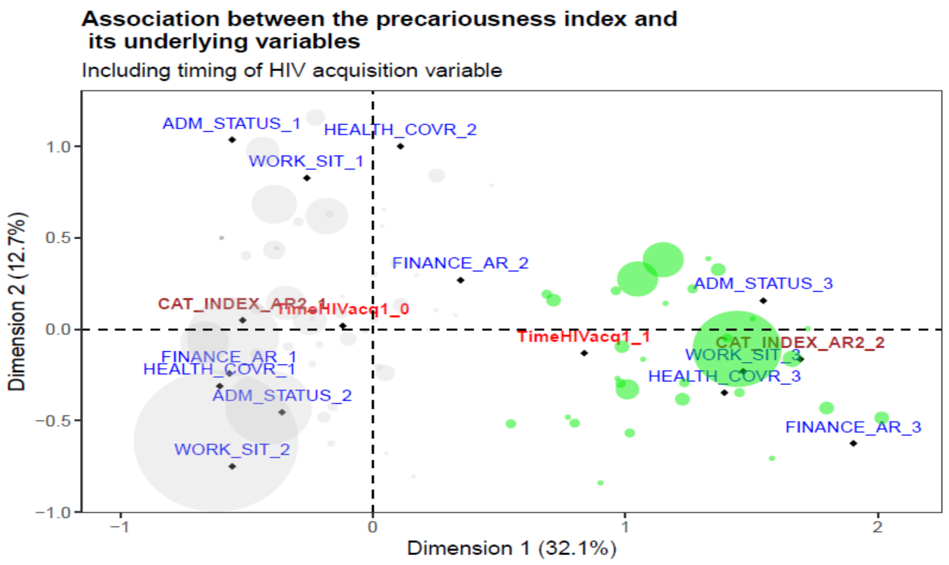

The last plot suggests that the third levels of the variables working situation (WORK_SIT_3), health coverage (HEALTH_COVR_3) are closely associated with each other. Moreover, these variables shown a strong relationship with the second level of the social disadvantage indocator (CAT_INDEX_AR2_2), which is linked to the acquisition of HIV during the first year in France (TimeHIVacq1_1).

Additionally, the first level of the social disadvantage indocator (CAT_INDEX_AR2_1) demonstrates an association with the first levels of the financial situation (FINANCE_AR_1) and health coverage (HEALTH_COVR_1) and is also strongly linked to the HIV acquisition after the first year in France (TimeHIVacq1_0).

Variables on participants' arrival conditions:

In addition to the indicator summarizing the precarious situation during the first 12 months in France, during the univariate analyses, we also identified a couple of additional variables that may play an important role in the acquisition of HIV after arrival in France. These variables are indicators of whether the participant felt compelled to leave their country of origin and whether the participant left their country of origin due to their sexual orientation.

These new variables provide valuable insights into the participants' experiences and circumstances, for this reason we performed an MCA including these three variables. The MCA plot is displayed in the figure below.

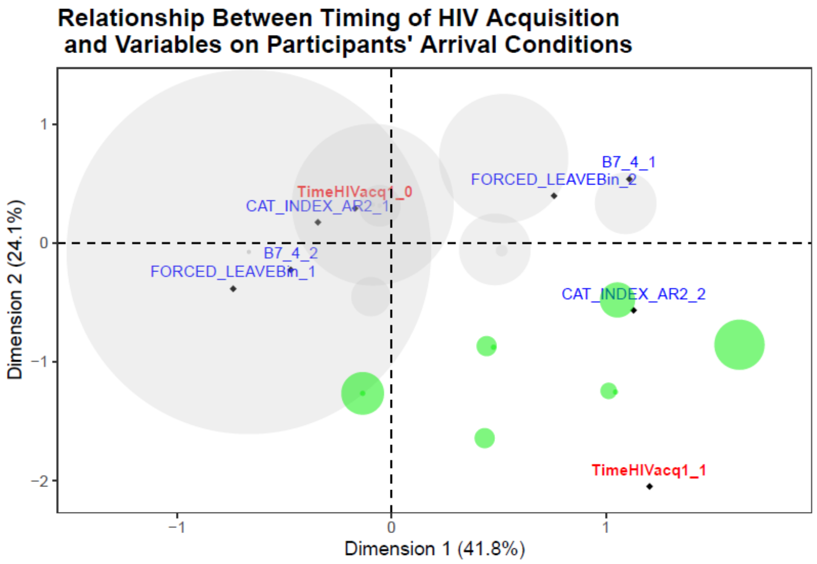

In the previous MCA plot, individuals with HIV acquisition during the first year after the arrival in France are represented by the green points, and the individuals who acquired HIV after the first year in France are in grey.

This analysis suggests a strong relationship between individuals who felt compelled to leave their birth country (B7_4_1) and those who left due to their sexual orientation (FORCED_LEAVEBin_2). Furthermore, there is a relationship between HIV acquisition during the first year in France (TimeHIVacq1_1) and the second level of the social disadvantage indocator (CAT_INDEX_AR2), which corresponds to individuals in a vulnerable situation.

Sexual behaviour variables:

A second group of variables that we identified as relevant during the univariate analysis are:

- SEX_PARTNERSBin: Number of sexual partners during the first year (categorized in two levels: 1 – more than 10 sexual partners, 0 - otherwise).
- CONDOM_USE2: Use of condoms with regular and occasional sexual partners (1 - Consistent use of condoms with all sexual partners, 2 - Use of condoms only with occasional partners, 3- Use of condoms only with the regular partners, 4 - No use of condoms at all, 0: Not concerned).
- MEET_SEXPART1: Frequentation of places for sexual encounters, saunas, dark rooms and outdoor cruising areas (1 - Yes, 2 - Not, 0 - Not concerned).
- MEET_SEXPART_C5: Use of dating apps to meet sexual partners (1 - Yes, 2 - Not, 0 - Not concerned).

The following MCA explores the relationship between these variables and the timing of HIV acquisition.

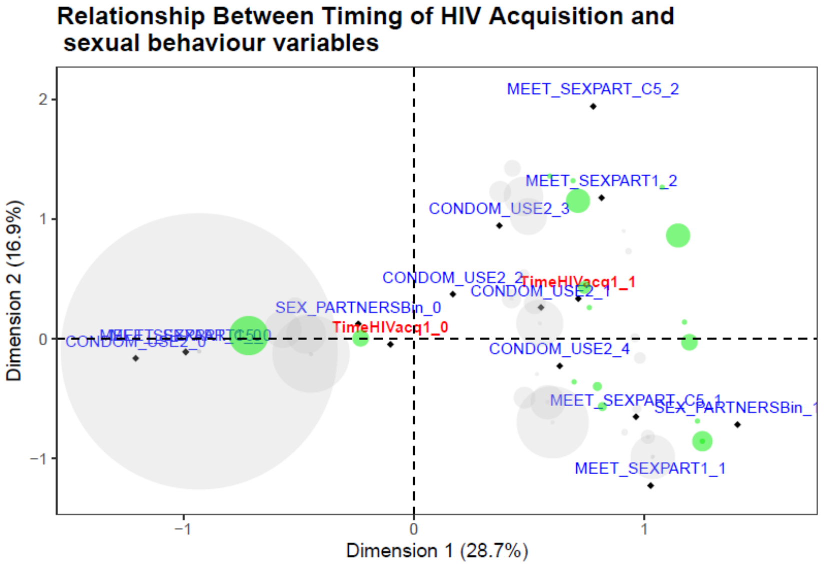

In the MCA plot, the variable "Use of condoms based on sexual partners" (CONDOM_USE2) shows a close relationship between its categories, suggesting that individuals tend to select similar categories in other variables. As a result, we decided to exclude this variable from our upcoming analysis.

Furthermore, our analysis reveals a significant correlation between individuals who frequent places such as saunas, dark rooms, and outdoor cruising areas for sexual encounters, and those who utilize dating apps to meet potential sexual partners. Therefore, in our next analysis, we will consolidate these two variables into a single variable.

Consequently, considering the outcomes of our last results, we conducted an MCA using a two-level variable: the number of sexual partners (1 - more than 10 sexual partners, 0 - otherwise) and the frequented places for sexual encounters, such as saunas, dark rooms, outdoor cruising areas, or the use of dating apps to meet sexual partners (1 - Yes, 2 - No, 0 - Not applicable). The resulting MCA plot is displayed below:

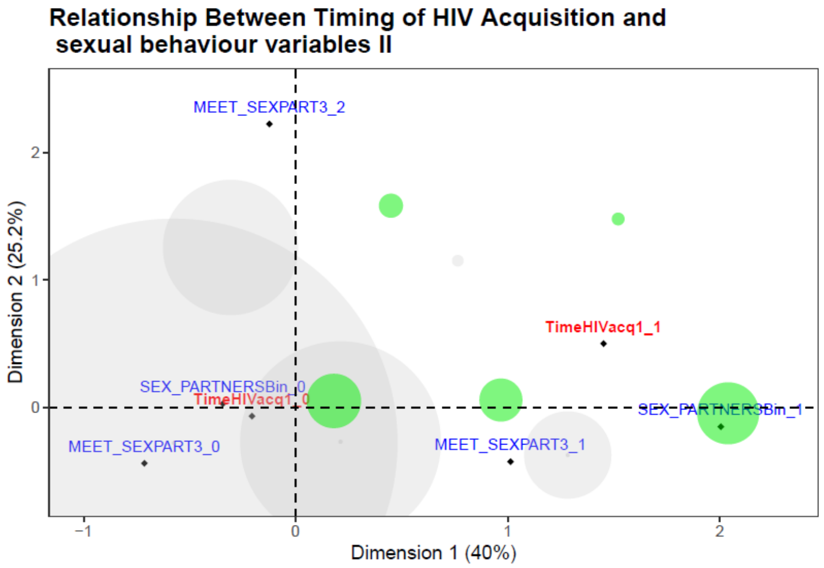

Based on the last MCA plot, we conclude that HIV acquisition during the first year in France is associated with having more than 10 sexual partners during the first year (SEX_PARTNERSBin_1) and frequenting places for sexual encounters such as saunas, dark rooms, and outdoor cruising areas, or using dating apps to meet sexual partners (MEET_SEXPART3_1).

Final Multiple Correspondence Analysis:

All the multiple correspondence analysis provided insights into the factors that may contribute to the early HIV acquisition after the arrival in France, but we must recall that this is a descriptive technique. The conclusions of these analysis were confirmed with logistic regression analysis whose results were included in the main document of this paper. The following MCA plot includes the categorical variables that were identified as relevant to characterize the HIV acquisition within the first year, according to the logistic regression model:

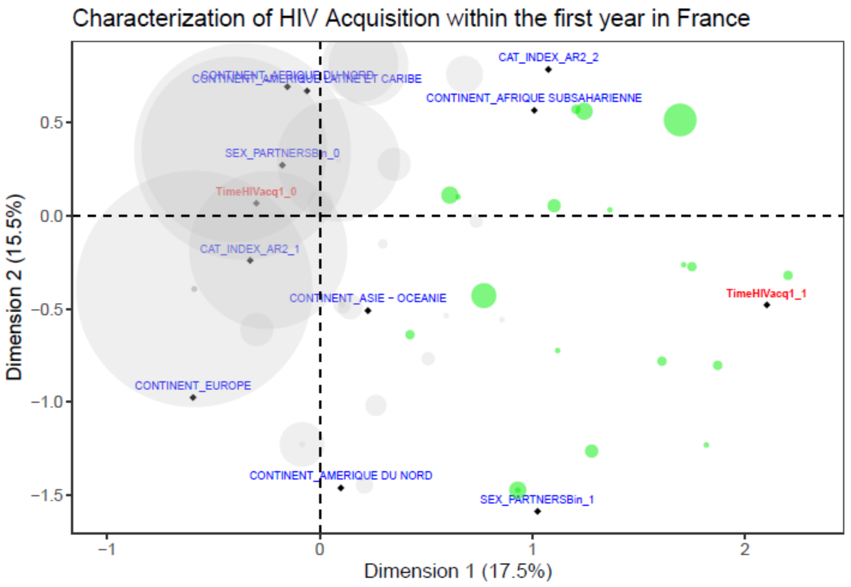

From the MCA plot we conclude that, HIV acquisition after the first year of the arrival in France is associated with the following categories:

- Lower values in social disadvantage index (CAT_INDEX_AR2_1), which refers to individuals with less vulnerable conditions during the first 12 months after the arrival in France.
- Having 10 or fewer sexual partners during the first year in France (SEX_PARTNERSBin_0).
- Individuals coming from Latin America and the Caribbean, North Africa, Asia – Oceania and Europe.

Individuals who do not belong to the categories above are more likely to acquire HIV during the first year.

References

1. *Joint estimation of CD4+ cell progression and survival in untreated individuals with HIV-1 infection.* **Mangal, Tara D and UNAIDS Working Group on CD4 Progression.** 2017, AIDS, pp. 1073-1082.

2. *Estimating Trends in Incidence, Time-to-Diagnosis and Undiagnosed Prevalence using a CD4-based Bayesian Back-calculation.* **Birrel, Paul J, et al.** 1, 2012, Statistical Communications in Infectious Diseases, Vol. 4.

3. *Bayesian back-calculation using a multi-state model with application to HIV.* **Sweeting, Michael J, De Angelis, Angela and Aalen, Odd O.** 2005, Statistics in Medicine, pp. 3991-4007.

4. *A universal data compression.* **Rissanen, Jorma.** 1983, IEEE Transactions on Information Theory, pp. 656-664.

5. *Variable Length Markov Chains.* **Bühlman, Peter and Wyner , Abraham.** 1999, The Annals of Statistics, pp. 480-513.

6. *A generalization of the PST algorithm: modeling the sparse nature of protein sequences.* **Leonardi, Florencia.** 2006, Bioinformatics, pp. 1302-1307.

7. *Context tree selection and linguistic rhythm retrieval from written texts.* **Galves, Antonio, et al.** 2012, The Annals of Applied Statistics, pp. 186-209.
